# Durable treatment response of relapsing CNS plasmacytoma using intrathecal chemotherapy, radiotherapy, and Daratumumab

**DOI:** 10.1002/ccr3.1451

**Published:** 2018-03-02

**Authors:** Ezzat Elhassadi, Maurice Murphy, Dayle Hacking, Michael Farrell

**Affiliations:** ^1^ Department of Haematology University Hospital Waterford Waterford Ireland; ^2^ Department of Pathology University Hospital Waterford Waterford Ireland; ^3^ Department of Radiotherapy UPMC Whitefield Cancer Centre Waterford Ireland; ^4^ Department of Radiology University Hospital Waterford Waterford Ireland

**Keywords:** CNS plasmacytoma, Daratumumab, extramedullary myeloma, intrathecal chemotherapy, radiotherapy

## Abstract

CNS myelomatous involvement is a rare complication of multiple myeloma with dismal outcome. This disease's optimal treatment is unclear. Combined approach of systemic therapy, radiotherapy, and intrathecal injections chemotherapy should be considered and autologous stem cell transplant consolidation is offered to eligible patients. The role of Daratumumab in this disease deserves further evaluation.

## Introduction

Multiple myeloma (MM) is a mature B‐cell neoplasm that accounts for approximately 1% of all cancers and slightly more than 10% of hematological malignancies in the United States (US) 1. A subset of MM patients develop extramedullary myeloma (EMM), defined by the presence of clonal plasma cells outside the bone marrow (BM) 2. EMM has been reported in 10–30% of patients 3, with prevalence increasing with prolonged disease course.

The presence of EMM at the time of diagnosis confers an adverse prognosis 4, 5. Extramedullary disease at relapse has a worse prognosis when compared with primary bone‐related plasmacytoma with an overall survival of <6 months 6. Central nervous system (CNS) involvement is rare and observed in 1% of MM patients. CNS EMM has an extremely poor prognosis with retrospective studies reporting a median overall survival of <6 months 7, 8.

Here, we present a challenging case of relapsed MM with CNS plasmacytoma treated with radiotherapy, intrathecal chemotherapy, and Daratumumab, which achieved a durable complete response.

## Case Report

**Figure 1:**
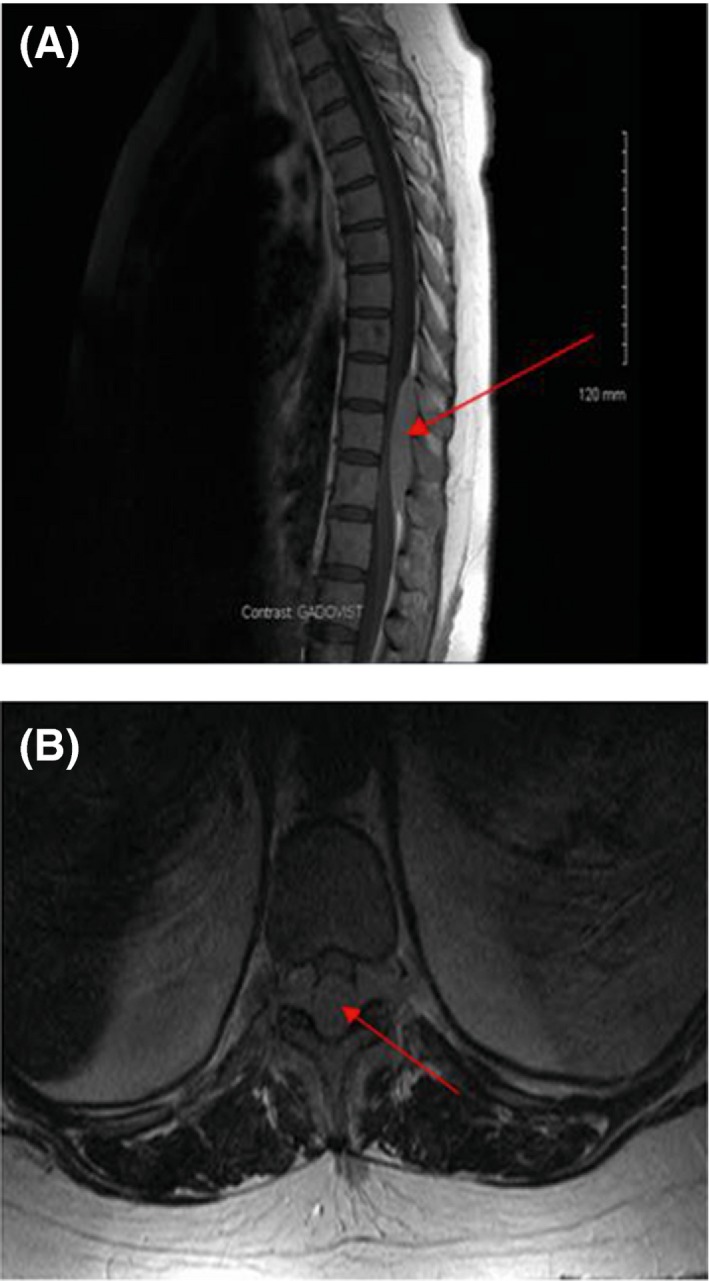
MRI thoracic spine showing posterior extradural extramedullary neoplastic mass (red arrows), extending from T10 to T12 (A, sagittal view; B, axial view).

**Figure 2:**
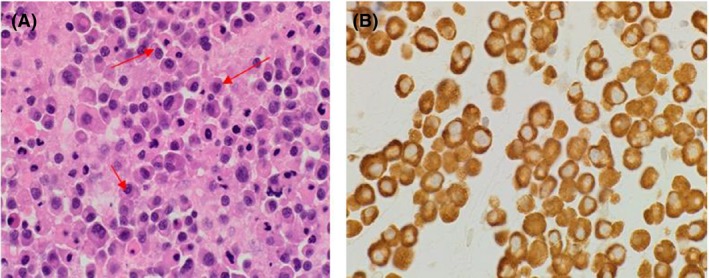
An extramedullary mass biopsy histology (A, H&E stain and B, Lambda light restriction) (Red arrows represent plasma cell infiltrate).

**Figure 3:**
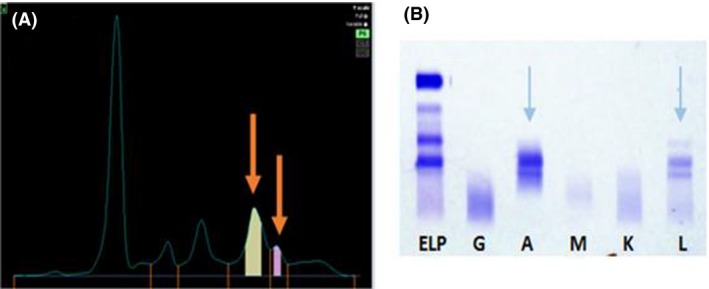
(A) Serum protein electropherogram (orange arrows = IgA monoclonal bands), (B) Immunofixation electropherogram (blue arrows = IgA band and lambda light chain restriction).

**Figure 4:**
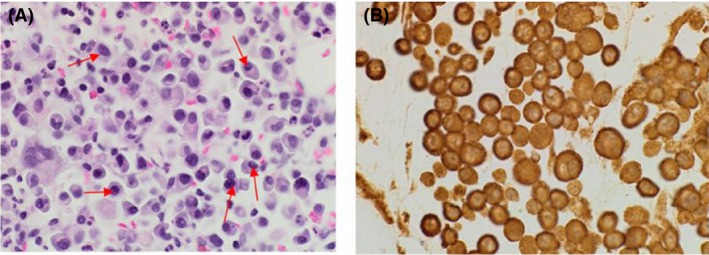
Bone marrow histology (A, H&E stain and B, Lambda light restriction) (Red arrows represent plasma cell infiltrate).

This 45‐year‐old woman presented with back pain in November 2013 and cross‐sectional imaging showed a posterior extramedullary extra‐axial thoracic spinal mass extending from T10–T12 (Fig. 1), which was surgically removed. Histology revealed sheets of neoplastic plasma cells which demonstrated immunohistochemical positivity for CD138, CD56, CD79a, and MUM‐1 protein with lambda light chain restriction (Fig. 2). She had an IgA lambda paraprotein band, and two bands were visible at the beta region of her serum protein electrophoresis sized 2.4 and 5.3 g/L, respectively (Fig. 3). The 24‐h urine protein was 0.63 g/L with a detectable lambda light chain restricted urine paraprotein of 0.40 g/L. Hemoglobin was 105 g/L, and creatinine and calcium were within their respective reference ranges. The BM aspirate was nondiagnostic, with poor quality making it unsuitable for FISH cytogenetics analysis. However, the BM biopsy was completely effaced by atypical plasma cells with lambda light chain restriction, consistent with a diagnosis of IgA lambda myeloma (Fig. 4).

She received four cycles of CyBord chemotherapy (cyclophosphamide 300 mg/m^2^ orally, weekly, bortezomib subcutaneous injection 1.5 mg/m^2^ weekly and dexamethasone 40 mg orally, weekly) every 28 days, after which her serum paraprotein was no longer detectable (near complete response, nCR). Antiviral, (Valtrex 500 mg once daily), antifungal mouth care (Mycostatin 1 mL QDS mouth wash), and pneumocystis prophylaxis (Septrin 960 mg BD Mon/Wed/Fri) were used throughout. Stem cells were harvested in March 2014, using high‐dose Cyclophosphamide (2 g/m^2^) with GCSF mobilization. Local radiotherapy was not delivered as originally planned as the patient underwent an autologous stem cell transplant (ASCT) with melphalan conditioning (200 mg/m^2^) in May 2014 and nCR was maintained.

She relapsed biochemically a year later and was treated with six cycles of RVD therapy (Lenalidomide 15 mg od orally every 21 days, bortezomib 1.3 mg/m^2^ every 2 weeks, dexamethasone 40 mg orally weekly), resulting in resolution of the paraprotein, with negative serum and urinary free light chains (nCR). Antiviral (Valtrex 500 mg once daily) and antifungal mouth care (Mycostatin 1 mL QDS mouth wash) were used, with Enoxaparin 40 mg subcutaneously once daily for venous thromboembolism (VTE) prophylaxis.

Lenalidomide and bortezomib therapy without dexamethasone were continued as maintenance treatment (five cycles in total) with the same supportive care. She developed a right popliteal deep vein thrombosis, despite VTE prophylaxis, requiring therapeutic anticoagulation, and Lenalidomide maintenance was discontinued. She remained on single‐agent bortezomib maintenance (1.3 mg/m^2^) every 2 weeks, and received six cycles in total.

**Figure 5:**
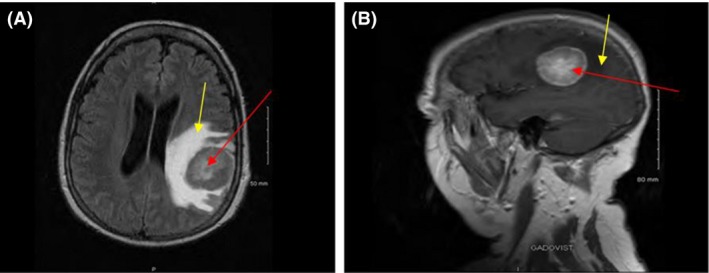
MRI brain showing a well‐circumscribed left parietal extra‐axial neoplastic mass (red arrows), with extensively associated vasogenic edema (yellow arrows) (A, axial view; B, sagittal view).

**Figure 6:**
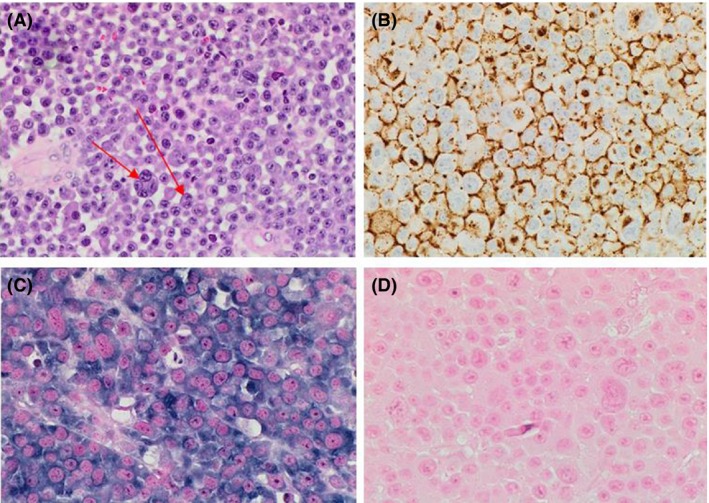
Brain mass biopsy histology. (A) H&E stain, (B), CD138 stain ×40, (C) Lambda light restriction (Positive), (D) Kappa light chain restriction (Negative). (Red arrows represent atypical multinucleated plasma cell infiltrate.)

In June 2016, she presented with disorientation, dysphasia, right‐sided weakness and pain behind her right eye without visual impairment. A brain magnetic resonance imaging (MRI) revealed a well‐circumscribed left parietal neoplastic mass (Fig. 5), with a negative spinal MRI. Neurosurgical biopsy demonstrated a lambda‐restricted plasmacytoma (Fig. 6). Immunophenotyping of the cerebrospinal fluid (CSF) revealed a population of CD38+/CD138+/CD19−/CD56+ plasma cells suggesting CSF infiltration with a clonal plasma cell phenotype. She exhibited no serum or urine paraprotein with normal serum kappa:lambda ratio, and her BM was uninvolved.

The patient commenced on pomalidomide (4 mg orally daily) and dexamethasone (40 mg orally weekly) with LMWH doses prophylaxis in 1st of July 2016. In addition, she received intrathecal injections (IT) of cytarabine (40 mg), methotrexate (12.5 mg), and hydrocortisone (20 mg) after which CSF clonal plasma cells were no longer detectable (5 IT injections in total). Concurrently, she received a craniospinal radiotherapy, 36 Gy to the brain in 20 fractions, 1.8 Gy each and 27 Gy to the spinal in 15 fractions.

The first week of pomalidomide therapy was complicated with cytopenia (nadir white cells 0.3 × 10^9^/L, neutrophils 0.07 × 10^9^/L, platelets 52 × 10^9^/L, and hemoglobin 84 g/L). Her course was further complicated by recurrent episodes of febrile neutropenia despite dose modification and GCSF support. In addition, she had significant gastro‐intestinal side effects despite appropriate antiemetics which necessitated stopping her pomalidomide therapy.

On 28 July 2016, A single‐agent Daratumumab therapy was commenced as per licensed schedule, 16 mg/kg intravenously, weekly cycle 1–8, every 2 weeks cycle 9–24, and then monthly from week 25 onward. Allopurinol 300 mg daily for 7 days was given in cycle 1 only, Valtrex 500 mg once daily, Mycostatin 1 mL QDS mouth wash, and Septrin 960 mg BD Mon/Wed/Fri were used throughout. Enoxaparin (40 mg subcutaneously once daily) continued as prophylaxis during all cycles.

The first 2 weeks of Daratumumab treatments were delivered as an inpatient with subsequent treatments administered in our day ward facility and is currently on week 68 of treatment. Daratumumab was well tolerated with some intermittent electrolytes disturbance. A low‐dose dexamethasone (4 mg TDS orally) was continued between cycles to treat the associated cerebral edema and tapered gradually with resolution of neurological symptoms. This combination therapeutic approach has resulted in complete resolution of her neurological signs and symptoms. Serial laboratory monitoring has shown no biochemical evidence of myeloma.

**Figure 7:**
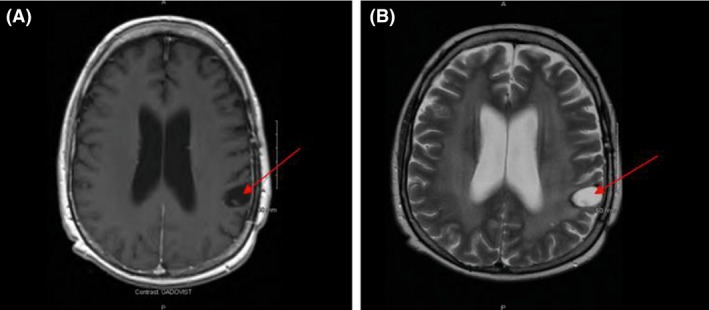
MRI brain showing residual small CSF filled cystic space at the site of previous neoplastic mass (Red arrows).

Figure 7 illustrates a follow‐up brain MRI scan performed on 12th of April 2017, revealing diffuse bilateral supra‐ and infra‐tentorial deep white matter changes in the brain consistent with the sequelae of whole brain radiation. There was a small CSF filled cystic space at the site of the previous extra‐axial left parietal mass and with no evidence of interval development of a new mass.

To our knowledge, this is the first case report describing the utilization of Daratumumab therapy in the management of CNS plasmacytoma in conjunction with craniospinal radiotherapy and IT triple therapy.

## Discussion

Intracranial plasmacytomas are rare and commonly a result of hematologic spread and to a lesser extent as a represent a direct extensions of myelomatous lesions of the skull or plasmacytoma involving the clivus or base of the skull 9. Retrospective studies highlight an extremely poor prognosis for CNS EMM with a median overall survival of <6 months despite treatment 7, 8, 10.

Extramedullary involvement, high‐risk cytogenetics, high lactate dehydrogenase (LDH) levels, and plasma cell leukemia at MM diagnosis were associated with an increased risk of CNS involvement 9. In this patient, although her LDH was normal at presentation, the Ig A MM subtype with extramedullary disease presentation and early relapse post‐ASCT confer a high‐risk disease phenotype. BM slides FISH cytogenetics analysis was attempted but failed due to low plasma cell count and poor slides quality. In addition, lack of radiotherapy consolidation to the initial spinal lesion, even though it was surgically removed, may have contributed to her later presentation.

Magnetic resonance imaging is the most sensitive imaging method for the detection of leptomeningeal infiltration. Cerebrospinal fluid analysis is mandatory including cytology, protein measurement, electrophoresis, and flow cytometry to detect clonal plasma cells. Surgical biopsy should be considered if the CSF analyses are not confirmative.

Optimal therapy for CNS myeloma is unclear. Most published reports to date describe dismal survival of 1–2 months with traditional approaches incorporating the use of IT chemotherapy and radiation. Alkylating agents penetrate the CSF poorly, and other conventional chemotherapies known to cross the blood–brain barrier (BBB) (high‐dose methotrexate or cytarabine) are not effective in treating MM. CNS EMM ideally should be treated with systemic therapy including drugs that cross the BBB and radiotherapy incorporated in any management plan especially in patient with neurological signs and symptoms. Intrathecal chemotherapy (IT) role in CN EMM management remains controversial 9. However, in this case, the presence of clonal plasma cell in CSF justifies the IT chemotherapy indication to optimize initial patient treatment.

Thalidomide and Lenalidomide in addition to high‐dose corticosteroids have also been reported to penetrate the BBB in nonhuman primates 11, 12. The third‐generation immunomodulator (IMiD), pomalidomide has demonstrated good penetrance of the BBB in a murine model 13. Notably, a durable CSF remission was recently reported using a pomalidomide‐dexamethasone treatment 14. Of course, the ability of IMiDs to treat CNS myeloma should be evaluated in a larger number of patients, ideally in the context of prospective trials. Pomalidomide‐dexamethasone therapy was commenced in our patient; however, pomalidomide was poorly tolerated and discontinued early as a result of prolonged cytopenia with recurrent infections and gastro‐intestinal upset.

To date, Marizonimb (Salinosporamide A) is the only known proteasome inhibitor that has the ability to cross the BBB, although evidence of clinical efficacy in CNS myeloma is lacking 15, 16. Survival advantage has been reported in CNS EMM patients with ASCT consolidation, and therefore, it should be offered to eligible patients 9.

Daratumumab is a humanized monoclonal antibody specific for CD38 [immunoglobulin (Ig)G_1_,*κ* subclass] that targets tumor cells *via* antibody‐dependent cell‐mediated cytotoxicity (ADCC), complement‐dependent cytotoxicity, and phagocytosis. Daratumumab may also initiate CD38‐mediated signal transduction leading to cell death 17.

Daratumumab was recently approved by the US FDA in November 2015 as a single‐agent treatment for patients with relapsed/refractory MM who have failed more than three lines of treatment regimens, including patients refractory to IMiDs and proteasome inhibitors. The efficacy of monoclonal antibodies in EMM has not been reported so far. In general, monoclonal antibodies do not cross the BBB if intact. Currently, there are no available data to suggest Daratumumab could cross the BBB; however, the fact that, the barrier can become more permeable in disease states and that there is no barrier in the meninges raises this possibility.

To our knowledge, this is the first report evaluating the efficacy of Daratumumab therapy in CNS plasmacytoma in combination with craniospinal radiotherapy and IT chemotherapy. Although the therapy of CNS EMM is difficult, the present case illustrates that a durable response (19 months) can be achieved by using systemic daratumumab therapy, IT chemotherapy, and craniospinal radiotherapy, which compares favorably to the reported literature (<6 months) 7, 8, 11.

Overall, in this patient, Daratumumab therapy showed a favorable tolerability profile without any serious adverse events. In this case, a part from electrolyte disturbances initially, requiring rigorous replacement, Daratumumab treatment was well tolerated and continues to be delivered in an outpatient setting. Serial MRI imaging follow‐up revealed continuous improvement in the CNS lesions, and the most recent brain MRI (20th of November 2017) showed a stable post‐treatment changes and no evidence of residual or recurrent disease.

## Conclusion

CNS plasmacytoma is an aggressive condition and, even with modern therapies, remains lethal with a median overall survival of only 4.6 months. However, in this case, the combined approach of craniospinal irradiation, triple‐therapy IT chemotherapy, and anti‐CD38 monoclonal antibody has produced a durable response to date, which far exceeds the reported data.

## Consent

Written informed consent was obtained from the patient for publication of this case report and any accompanying images. A copy of the written consent is available for review by the editor‐in‐chief of this journal.

## Authorship

EE: involved in the medical care of the patient and wrote the manuscript. MM: provided the diagnostic histology materials. DH: provided the radiotherapy treatment and involved in drafting the manuscript. MF: provided imaging materials and involved in drafting the manuscript. All authors approved the final version of the manuscript.

## Conflict of Interest

None declared.
